# A Rare Case of Small Bowel Obstruction due to Migration of a Percutaneous Biliary Stent

**DOI:** 10.1177/23247096241238527

**Published:** 2024-04-22

**Authors:** Luca Ghirardelli, Aldo Alberto Beneduce, Simone Gusmini

**Affiliations:** 1 IRCCS San Raffaele Scientific Institute Milan, Italy

**Keywords:** biliary stent, small bowel obstruction, biliary leak, biliary stent migration, biliary stent complication, percutaneous biliary stent

## Abstract

Biliary endoprostheses are widely used in the treatment of biliary lithiasis, malignant and benign strictures, and occasionally in long-lasting biliary fistulas. They can be placed endoscopically during endoscopic retrograde cholangiopancreatography and radiologically (percutaneous) when the endoscopic route is not feasible. Complications associated with the endoscopic placement of biliary endoprostheses are well described in the literature, with migration being the most common. Intestinal obstruction is a rare complication associated with the migration of these devices. There are no reports in the literature of this complication occurring after percutaneous placement. We present a case of a patient who arrived at the emergency department with ileal obstruction secondary to the migration and concurrent embedding of a covered stent placed radiologically to treat a biliary leak after surgery. The patient underwent diagnostic laparoscopic and ileal resection, revealing a lithiasic concretion at the tip of the stent, causing the small bowel obstruction.

## Introduction

Biliary endoprostheses are used for treating both benign and malignant strictures of the bile ducts,^1^ and occasionally in cases of iatrogenic biliary fistulas.^2^ These can be inserted during endoscopic retrograde cholangiopancreatography (ERCP) or radiologically when the endoscopic route is not feasible, for instance, after gastrointestinal surgeries. Complications associated with endoprosthesis placement are well documented in the literature, with the most common being proximal and distal migration, occurring at an incidence of 5% to 10%.^3^ Risk factors for migration include prolonged endoprosthesis placement (more than 1 month), endoprosthesis diameter >10 mm, sphincterotomy, the use of plastic stents (PS), and bile duct diameter >10 mm^3^. Often, migration does not result in clinical problems, and the prosthetic material is expelled from the intestine. Occasionally, it can lead to intestinal perforation, especially if the stent is non-plastic.^4^ Very rarely, stent migration manifests solely as intestinal obstruction. Furthermore, there are few reports in the literature regarding complications associated with the percutaneous placement of endoprostheses.

## Case

**Figure 1.**
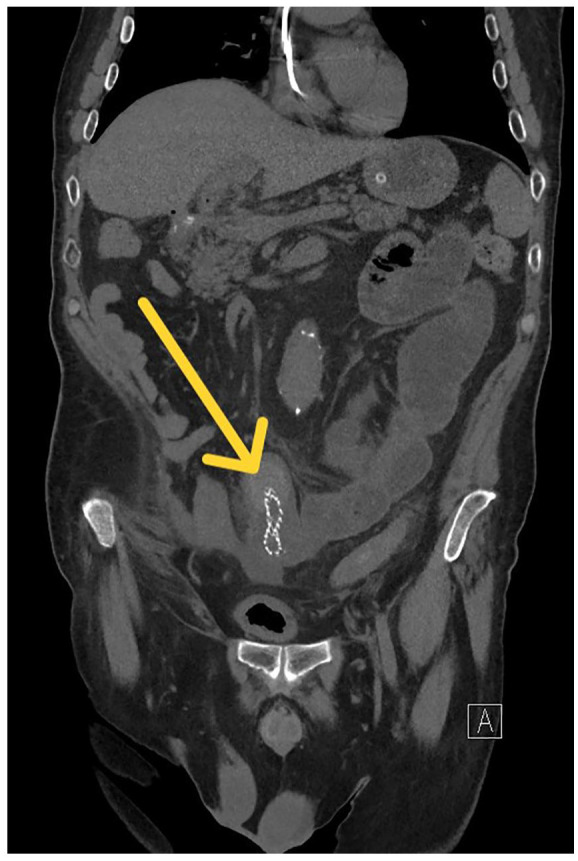
CT scan revealing small bowel obstruction and the migrated stent (arrow).

**Figure 2.**
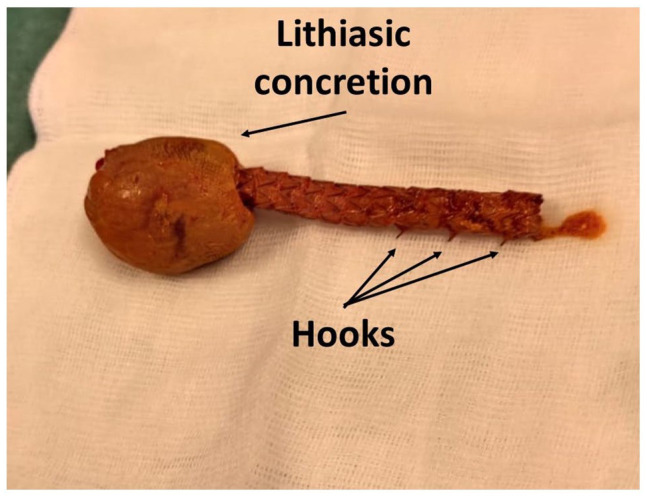
Extracted stent with hooks and lithiasic concretion in evidence (arrows).

We report the case of a 65-year-old man who presented to the emergency department with abdominal pain (Numerical Rating Scale 5), vomiting, and constipation for approximately 48 hours. The patient denied having a fever. Upon evaluation, the patient displayed marked abdominal distension associated with metallic bowel sounds. No signs of tenderness were noted. In his medical history, a gastro-duodenal resection and cholecystectomy with Roux-en-Y reconstruction were performed 12 months prior due to a bleeding and perforated duodenal ulcer (cholecystectomy was performed for gallbladder involvement). Postoperatively, the abdominal drain reveals a cystic stump fistula (Strabergh type A biliary duct leak) that occurred on the seventh postoperative day. During admission, findings included coronary stenosis requiring percutaneous angioplasty, a ventricular thrombus, and renal calculi contributing to chronic kidney disease (CKD). Due to the delayed manifestation of biliary fistula, the patient was initially treated with external-internal biliary drainage and maintaining the abdominal drainage, which was removed after 20 days due to the resolution of the output. As a persistent cystic stump leak was noted on radiological follow-ups (5 weeks after the index operation) in a patient with high surgical risk due to comorbidities, a decision was made to radiologically place a fully covered self-expandable metal stent (FCSEMS) (covered GORE biliary endoprosthesis, Viabil, 10 mm diameter, and 10 cm length), resolving the leak. In the emergency department, blood tests revealed a white blood cell count of 12.6 × 10^9^/L (r.i. 4.8-10.8), an increased LDH level of 320 U/L (r.i. 125-220), and a C-reactive Protein level of 44.1 mg/L (r.i. <6.0). Abdominal X-ray showed significant distension of jejuno-ileal loops with air-fluid levels, along with a radiopaque object consistent with the known prosthesis in the right iliac fossa. After nasogastric tube placement, a non-contrast abdominal computed tomography (CT) scan (serum creatinine, 2.5 mg/dL) demonstrated small bowel obstruction (SBO) secondary to stent migration (Figure 1). The stent showed material resembling a biliary concretion at its distal end. After completing preoperative assessments and anesthesia evaluation, the patient underwent diagnostic laparoscopic surgery. During the operation, SBO was confirmed due to the presence of an endoluminal foreign body partially embedded in the wall of the ileum. As intestinal resection was necessary, the procedure was converted to a mini-laparotomy with ileal resection and creation of a manual end-to-end ileo-ileal anastomosis. Examination of the surgical specimen confirmed the presence of the biliary endoprosthesis, at the distal end of which a lithiasic concretion causing intestinal obstruction had formed (Figure 2). Neither the CT scan nor the surgical intervention revealed a collection at the site of the previous fistula, confirming the resolution of the biliary fistula problem. The subsequent postoperative course proceeded without complications, and the patient was discharged on the fifth postoperative day.

## Discussion

Biliary stenting is a well-established procedure for managing pancreatic, gallbladder, and liver diseases, often performed during endoscopy.^2^ In this specific case, ERCP was not possible due to the patient’s Y-en-Roux reconstruction following gastro-duodenal resection. Therefore, a percutaneous approach was chosen to treat the leak. Several randomized controlled trials (RCTs) have proven that PS are superior to sphincterotomy alone in treating low-grade biliary duct lesions (Strasberg A-B).^5,6^ However, in cases of post-cholecystectomy bile leaks persisting after the insertion of a single PS, FCSEMSs were found to be superior to PS in a comparative non-randomized study.^7^ In our patient, who had previously undergone internal-external biliary drainage (comparable to PS in terms of efficacy), a decision was made to place an FCSEMS after 5 weeks of persistent biliary leak, leading to complete resolution. Complications associated with the placement of biliary endoprostheses are well documented in the literature and include stent migration, cholangitis, stent blockage, hemorrhage, perforation, and pancreatitis. Among these, stent migration is the most common complication, occurring in 5% to 10% of patients with biliary stents.^3^ Proximal migration of the stent up the biliary tree may result in biliary obstruction or be asymptomatic. Risk factors include short- or large-diameter stents. Distal migration is usually asymptomatic as stents pass through the intestines without problems; in fact, up to 86% of ingested foreign bodies traverse the intestinal tract.^8^ Known risk factors for distal migration include large-diameter stents, straight-type stents, stent duration >1 month, common bile duct diameter >10 mm, sphincterotomy, use of PS, and stenting of benign lesions.^3^ It is hypothesized that malignant biliary lesions anchor the stent, preventing downstream dislodgement. Intestinal obstruction is an extremely rare complication, with very few cases reported in the literature, and none were secondary to the migration of a percutaneous placed FCSEMS which is unlikely to migrate due to the presence of hooks at the proximal end. Likely, these same hooks anchor themselves to the walls of the intestine, increasing the risk of occlusion and perforation. Patient-specific risk factors for obstruction include postsurgical adhesions, diverticular disease, and hernias.^9^ Our patient had several risk factors for stent migration and obstruction. Computed tomography scan has proved superior diagnostic capabilities for SBO with a sensitivity and specificity of 91% and 89%, respectively. Computed tomography scan, especially enhanced contrast CT scan when feasible, is useful for treatment planning (conservative vs surgical) and selecting the surgical technique (laparoscopy vs laparotomy).^10^ Multiple intriguing case reports in the literature document successful endoscopic management, as well as alternative approaches such as resection, abscess drainage, enterotomy/colotomy, or a combination thereof, in patients presenting with perforation, abscesses, and obstruction.^11-13^ Most distally migrated stents can be managed endoscopically, such as extraction by operative colonoscopy if migrated into the large bowel. When stents migrate into the small bowel and do not respond to conservative management, surgical intervention is necessary. Laparoscopy is widely accepted as the first approach in SBO, especially when resection is not planned.^14^ In our case, the use of laparoscopy confirmed the suspected diagnosis and avoided a large laparotomy, leading to a faster recovery after surgery. In conclusion, intestinal obstruction is a rare complication of biliary endoprosthesis migration. In patients with risk factors for obstructive complications and an expected stent duration of >1 month, the use of PS is preferable, when possible, despite the higher risk of migration. This is likely because PS carries a lower risk of intestinal obstruction compared with the FCSEMS being used. When PS is not applicable (as in our case) or the treatment of biliary leak with PS fails, the use of FCSEMS is necessary. In such cases, if technically feasible, it would be advisable to remove the stent after leak resolution. For the diagnosis of this rare condition, a thorough analysis of the patient’s medical history is crucial, although CT scan proves high sensitivity and specificity, especially in the presence of a metallic prosthesis. Currently, surgery (laparoscopic or laparotomic) remains the therapeutic option of choice in cases of migration and obstruction in the small intestine, although cases of extraction using endoscopic methods have been described.^15^
